# Newly Graduated Nurses' Experiences of Taking Responsibility for Safe Nursing Care in Their First Year in Connection to COVID‐19: A Qualitative Study

**DOI:** 10.1002/nop2.70530

**Published:** 2026-04-08

**Authors:** Ann‐Catrin Blomberg, Birgitta Bisholt

**Affiliations:** ^1^ Department of Health Sciences Karlstad University Karlstad Sweden; ^2^ Lovisenberg Diaconal University College Oslo Norway; ^3^ Institution of Health Sciences Red Cross University Stockholm Sweden

**Keywords:** COVID‐19, experience, graduate nurses, professional development, responsibility, safe nursing care

## Abstract

**Aim:**

To describe newly graduated nurses' experiences of taking responsibility to ensure safe nursing care in their first year and how COVID‐19 affected their professional development.

**Background:**

Newly graduated nurses faced many challenges and great pressure from healthcare to take responsibility to ensure safe nursing care under their first year in the profession. Challenges increased with COVID‐19, possibly affecting safe nursing care and the psychosocial work environment.

**Design:**

A descriptive qualitative design was chosen and data were collected through group interviews from nurses in their first year as newly graduated nurses (*n* = 15) in the middle of Sweden. Semi‐structured interviews were conducted digitally, and inductive thematic analysis was used to analyse the data.

**Results:**

The experiences of newly graduated nurses were summarized along two themes: *Own responsibility from start* and *Challenges lead to professional development*. The participants described how they initially had to take full responsibility for patient's care. They described feelings of inadequacy and uncertainty in relation to safe nursing care and highlighted a lack of support from more experienced colleagues. They had to take their own initiative more, conduct complex caregiving, and had the opportunity to share experiences with other professions. The pandemic contributed to increased demands on their own responsibility for patient's care. Experiences were both positive and negative, and different challenges led to professional development.

**Conclusion:**

Newly graduated nurses felt that challenges and responsibility led to their development in the profession. Support and competence development are needed in their first year.

**Implications for the Profession and/or Patient Care:**

Newly graduated nurses should be offered continuous support and well‐organized competence development to enhance professional self‐confidence. Well‐functioning teams create a sense of belonging, where knowledge and experience are exchanged, leading to professional development.

**Patient or Public Contribution:**

There was no patient or public contribution.

## 1 Introduction

This study describes newly graduated nurses (NGNs') experiences in their first year in the profession when having to take responsibility for safe nursing care. Registered nurses (RNs) face many challenges and much pressure due to the generational change of personnel (high average age), ongoing medical technology development, increased average lifespan among the population, and increased demands of patients for participation in their care (Buchan et al. 2019; SFS 2014:821). It is expected according to Buchan et al. (2019) that the retention of NGNs continues to be problematic. Providing high quality and safe nursing care now and going forward means securing long‐term staffing with the relevant competence to meet future care needs. These challenges assume that NGNs have the competence required to perform their professional tasks immediately after nursing education completion. NGNs require continuous competence development throughout their professional life (Lindfors et al. 2022). An increased workload in connection with COVID‐19 brought additional challenges for NGNs in the form of having to take responsibility for seriously ill patients and having to make their own priorities without the support of experienced RNs, which may have affected safe nursing care and the psychosocial work environment (Sveriges Kommuner och Regioner [Sweden's Municipalities and Regions] 2021). For that reason, it is of interest to examine how NGNs describe their experiences of their first year as a NGN in connection to COVID‐19.

## 2 Background

RNs belong to a profession which, according to Bentling and Jonsson (2010), entails a social responsibility, to possess important knowledge, skills and abilities, and to plan care based on evidence. This is based on “knowing that” and “knowing how”, specific tasks should be done and “knowing why”, which involves taking responsibility for what is right and wrong in the current situation. To prevent failure or safe nursing care being overridden, the value of following established routines and methods is asserted (Bisholt 2009, 2012).

World Health Organization (WHO) safe nursing care focuses on a systemic approach to prevent avoidable harm, integrating strong leadership, a safety culture, skilled staff, teamwork, technology, and patient engagement to create safer systems, processes, and environments, ensuring nurses have the right skills, resources, and support to deliver high‐quality (World Health Organization 2023). Safe nursing care, as defined by Environmental Sciences, depends on nurses having strong interpersonal, communication, and management skills, as well as empathy and emotional intelligence, to ensure successful teamwork and enable them to confidently make decisions and solve problems in complex healthcare situations in clinical practice (Vaismoradi et al. 2012). This also involves nurse managers' responsibilities, such as coordinating and integrating aspects of quality care and dealing with unsafe practices (Rashvand et al. 2017).

The profession is governed by a code of ethics and obliges the RNs to show humanity and respect, to safeguard the patients' interest and promote the patients' right to self‐determination. It also includes scientific advances involving care robots or the like, where the nurse must ensure that technology does not replace human relationships (International Council for Nurses 2013). Forsberg (2016) believes that professional identity is not only created within us through reflection, but in dialogue with other professions. However, the definition of competence is inconsistent, has changed over time and varies in different countries (Gunawan et al. 2020). In this study, the meaning of competence is based on Ellström's (1992) definition to act and perform the specific duty in a certain situation and to reflect on and critically analyse and evaluate one's way of carrying out the work. It is not only made up of education and professional experiences but also integrates theory and practice. Nursing competence includes three dimensions according Jormsri et al. (2005): Clinical competence—clinical skills, assessment, intervention and judgement. General competence—communication, critical thinking and problem‐solving. Moral competence—practicing while maintaining morals and responsibilities within one's practice. This is because nursing practice depends not only on technical knowledge and skills but also on values, beliefs and ethics, which play a significant role in shaping their decision.

RNs' professional responsibility entails (Socialstyrelsen [The National Board of Health and Welfare] 2017) that RNs are responsible for and lead nursing work. This means possessing the competence to be able to establish a trusting relationship with the patient and relatives and, in collaboration with the team, be able to assess, plan, implement, and evaluate nursing interventions to ensure patient's safety. According to Wallinvirta (2011), this responsibility includes a formal responsibility, where principles, guidelines, and practical rules dominate, and an overall responsibility in relation to organizations and society. It also includes a personal responsibility containing a moral requirement to choose actions to care for the patient based on an ethical approach. In Sweden, nursing education is 3 years (180 ECTS credits) and leads to both a professional degree and a bachelor's degree. After that, there is the option of choosing a specialist education that leads to a master's degree (60 ECTS credits). There is a great need for nurses, and according to (Sveriges Kommuner och Regioner [Sweden's Municipalities and Regions] 2021), demand is greater than supply. Within healthcare, NGN in most regions are offered competence development and the opportunity to study independent courses at university level. Education and informal learning are considered essential to develop individual learning (Ellström 1992).

Internationally, show that during their first year, 18%–50% of NGNs´ chose to change jobs or even quit their employment (Aiken et al. 2017; Wu et al. 2012). Even in Sweden, Rudman et al. (2014) found that every fifth RNs change jobs. Also a result of COVID‐19, led to a decrease in the number of experienced RNs in recent years, with some choosing to quit due to anxiety, social problems and post‐traumatic stress (Melander et al. 2024). Healthcare thus has difficulties in recruiting, employing and retaining experienced RNs and NGNs (Sveriges Kommuner och Regioner [Sweden's Municipalities and Regions] 2021). The first years in the profession are a critical period and NGNs can find themselves in complex situations, where patient safety is required to prevent the patient from being exposed to a care injury (Rudman et al. 2020; SFS 2010:659). There is a risk that NGNs feel inadequate, assume responsibility they are not prepared for and are expected to quickly become competent professionals (Casia et al. 2025; Gellerstedt et al. 2019). Therefore, this study aims to describe NGNs' experiences of taking responsibility for safe nursing care during their first year in the profession and how COVID‐19 affected their professional development.

## 3 Methods

### 3.1 Design

A descriptive qualitative design was used to gain an understanding of NGNs' experiences of taking responsibility for safe nursing care and how COVID‐19 affected their professional development. Data collected using semi‐structured group interviews were analysed using inductive thematic analysis (Braun and Clarke 2006, 2021). This study is in line with the COREQ criteria for reporting qualitative studies (Tong et al. 2007).

### 3.2 Setting

This study was conducted in a central region in Sweden. The NGNs had worked as NGNs for nearly 1 year and represented both central and district hospitals. These three hospitals provide both emergency and planned treatment in several specialized fields and have approximately 500 hospital beds. The programme started in 2016 and is given to NGNs during their first year of employment. The content of the programme covers three areas: the nurse's profession and leading nursing work, skills training at the clinical training centre, quality and patient safety. The programme's structure consists of individual as well as team training and lectures. A total of 10 mandatory occasions (10 working days) are included in the programme. The program is mandatory for all NGNs within the region irrespective of care speciality. It is administered from the region's educational centre and the leader, employed by the region, is the director of studies.

### 3.3 Participants and Data Collection

The study's inclusion criteria were NGNs who began to work 1 month after he outbreak of the COVID‐19 pandemic. In the transition from education to professional work, all NGNs participated in a 12‐month Clinical Development Programme (KUP I) in their healthcare region and were in the end of the programme. A convenience sampling strategy was used to recruit NGNs, who could provide insights from their experiences. A request for participation was sent by the director of studies within the region to 46 NGNs via e‐mail and 15 NGNs agreed to participate. There were 12 women and three men of varying ages between 23 and 48 years (average = 30.2, median = 26). Some had previous work experience from healthcare. Others from other professions. Most came straight from high school to university studies, while some had previous education at university level. Their current employment was within somatic inpatient care such as medicine, surgery or emergency care at a county hospital. The participants had a minimum of nine and a maximum of 10 months' experience working as a NGN. In this study, means NGNs with less than 1 year experience as a RN.

A semi‐structured interview guide was used during the group interviews, which were conducted digitally using Zoom between November and December 2020. Online data collection generates data at a level comparable to in‐person focus groups (Richard et al. 2020). The interview guide was developed by both authors and discussed with the region director of studies, to ensure that information relevant to the aim of the study could be collected. A pilot interview was conducted to validate the interview guide, and it was not included in the study. No changes to the interview guide were made. The main questions were as follows: Can you tell us what it is like to care for patients as an NGN? What has it been like to work as a nurse during the pandemic? How do you handle care situations that are more demanding? What support have you received in these situations?

Conducting interviews digitally made it difficult to gather the group because the participants had different shifts in different hospitals and it was during the ongoing COVID‐19 pandemic. It may also have been perceived as safer to participate from their own home or another location of their choosing. The director of studies booked time for interviews with the NGNs by personal contact with each one. Totally five interviews were conducted and the group sizes varied between six—one participant (Kvale 1996). The reason why there was only one participant in one of the groups was illness and late cancellation. Both authors participated during the interviews and introduced themselves and the reason for the study, one interviewed and the other observed and listened and came up with reflective questions. Each interview ended with a summary, where the participants confirmed that it was understood correctly. In this study, the authors were not dependent on the participants, and the participants were not involved in the interpretations or conclusions. The interviews lasted between 37 and 75 min (average 56 min) and were recorded and transcribed verbatim.

### 3.4 Analysis

The qualitative data were analysed using inductive thematic analysis (Braun and Clarke 2006, 2021) to build an understanding of NGNs' experiences of caring for and ensuring patient care in their first year. The individual interview followed the analysis process in a similar way to the group interviews. Data were thus analysed using inductive and thematic analysis to identify codes, sub‐themes, and themes. Initially, the authors read and reread the transcripts separately several times. Then they read them together to gain a deeper understanding of the data. Preliminary codes were generated from the data after familiarising with the dataset, and data for similar codes were collated. The next phase involved examining the codes to develop significant patterns of meaning within the dataset, which are referred to as potential sub‐themes. Next, meaning units were highlighted and patterns were noted that addressed the aim of the study. Ideas and suggestions were noted. The ideas and suggestions identified six codes, and they were analysed and grouped according to similarities for sub‐themes and tentative themes. The development of themes was an interactive process, with themes being reorganised and refined as the phases of the analysis progressed. Finally, after the authors' discussion, the preliminary themes were re‐examined and further refined until consensus was reached. The analysis process was iterative and reflexive to promote a richer and more nuanced understanding of the data. In order to meet the reflexivity, regular, joint discussions have been held, and continuous reflection has been carried out during the analysis process. The data were analysed interactively and thereby we gained insight into our significant role in interpretation and theme construction. This added depth and rigour to the data analysis by considering our individual perspectives on the interpretation and theme construction (Braun and Clarke 2006, 2021).

To ensure rigour, one of the authors, who had previous experience with group interviews, served as an observer. It is important to note that the moderator has the power to guide the discussion and influence interaction within the group. During the group interview, the moderator allowed the participants to speak freely but ensured that the content stayed within the aim of the study. After each interview, a summary of what had emerged was produced, and the participants were asked to confirm that it had been understood correctly. This was done to ensure that the authors' preconceptions had not influenced the results. The analysis process was interactive and reflexive. Both authors have processed the material individually, as well as through joint discussions and continuous reflections throughout the analysis process. This also meant that our preconceptions were constantly questioned.

### 3.5 Ethical Consideration

The study was conducted according to The Code of Research Ethics as expressed in the latest version of the Helsinki declaration (World Medical Association 2024). The operations managers of each clinic at the hospitals gave their permission to conduct the study. The NGNs received written and verbal information that participation was voluntary, and that they could withdraw at any time without any consequence for their employment. Informed consent was obtained from all NGNs before the interviews by the director of studies. The study was given ethical approval by the Research Ethics Review Board ‘REDACTED’. In accordance with the European Union General Data Protection (GDPR), data were stored on the servers of one university and protected by the firewalls.

## 4 Results

**FIGURE 1:**
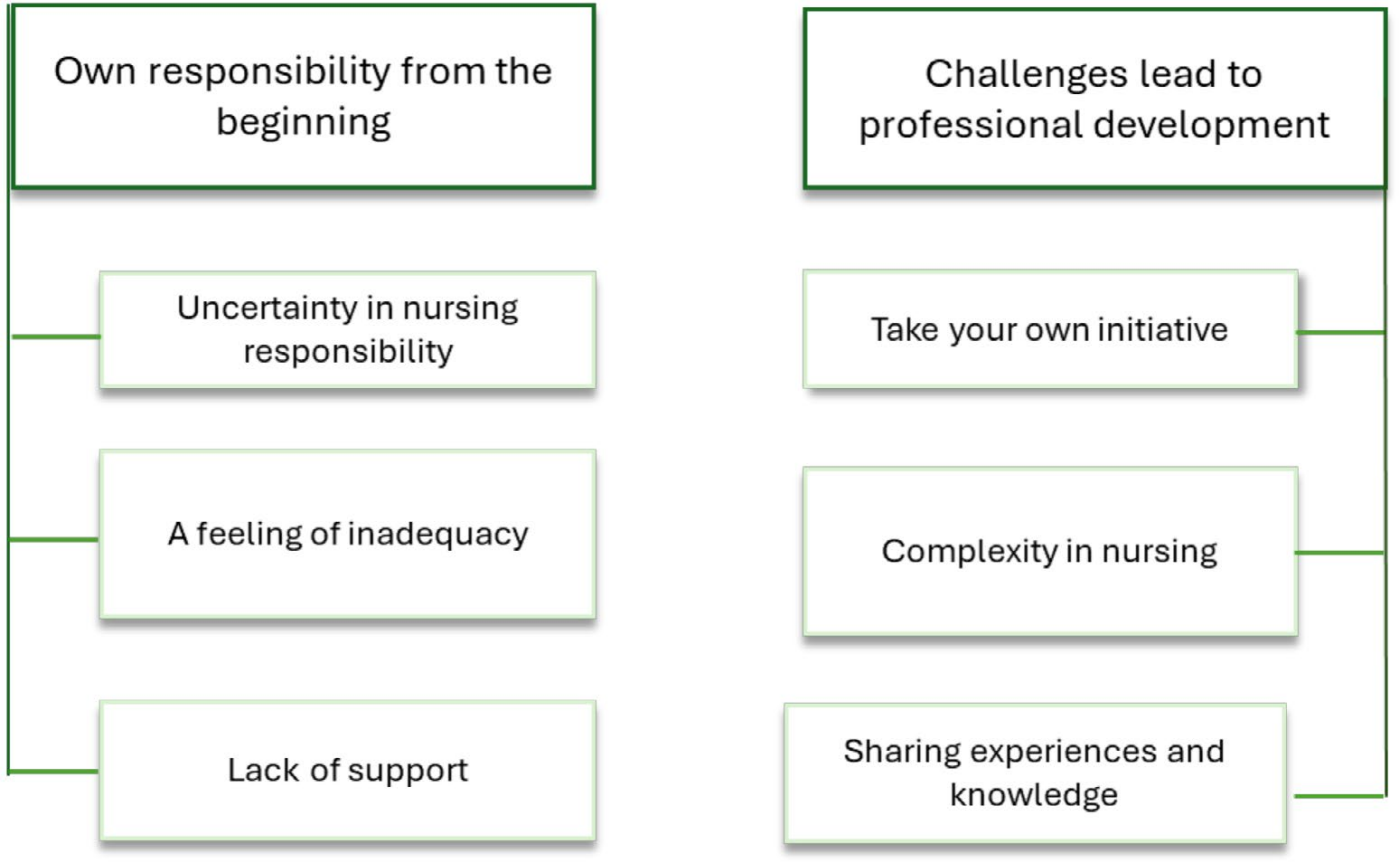
Thematic map presenting themes and sub‐themes.

The analysis resulted in two themes: *Own responsibility from the beginning and Challenges lead to professional development* with associated subthemes that describe NGNs' experiences during their first year in the profession and how COVID‐19 affected their professional development (Figure 1).

### 4.1 Own Responsibility From the Beginning

The NGNs described that right from the beginning they had to take personal nursing responsibility for the patient. They described experiences of uncertainty when it came to different priorities and assessments of the patient's need for care. Most often, they were responsible for assessing the care needs of several patients, and the requirement for prioritization increased significantly when caring for more care‐demanding patients. If something unexpected happened, their planning fell apart and led to the re‐prioritization of the work. The NGNs emphasized that they did not always have an experienced colleague to consult when it came to different priorities. In connection with COVID‐19, uncertainty increased when they were also responsible for other patient groups and seriously ill patients with whom they had no previous clinical experience. There was a lack of routines and unclear hygienic guidelines which could also vary over time. During certain shifts, only NGNs were present, and they were forced to make decisions that they were not considered competent to make. NGNs felt that their uncertainty could affect patient safety.

#### 4.1.1 Uncertainty in Nursing Responsibility

The NGNs described experiences of uncertainty in prioritizing and assessing patient care needs and above all when they had nursing responsibility for several patients. It also emerged that the responsibility for medical technology was considered demanding and there was uncertainty when handling and dosing medicines when there were no experienced colleagues to consult. In emergency situations, the NGNs described that they took responsibility in the situation and contacted other professions to be able to guarantee safe nursing care.

I can only agree with xx there really, that you have done things that you might not be sure about…. I we collaborate a lot in our staff group, and it is difficult to make a further assessment, when I don't have much experience…. as well as how we should dose medicines… a lot of pumps, pain pumps and it's very technical… that's what we all think is demanding of responsibility and that you've had to step up and have to take so much responsibility (5).

The NGNs was graduated in connection with the outbreak of the COVID‐19 pandemic. It was not only a challenge to be new to the profession, but also new to a pandemic. There were new challenges, and the participants described the confusion about unclear hygiene guidelines that changed over time. Suddenly they would be responsible for patient groups they had no previous experience of and serve in transit wards for patients with COVID‐19. Early on, they had to take their own nursing responsibility for planning patients´ care and this resulted in uncertainty in carrying out nursing activities without supervision.

…we graduated almost as the pandemic began, which has made me think it has been a challenge in several ways. Not just being new, but also being new in a pandemic, where suddenly everyone is new because no one really knows how everything is going to work and so on. So that I think it has been an extra test for us who have just finished in January. There have been many new tests and routines that have changed every other day, which you then must try to learn as new (5).

#### 4.1.2 A Feeling of Inadequacy

Several of the NGNs experienced a feeling of inadequacy when they were responsible for multi‐morbid patients. There was a fear that patients did not receive the care they believed the patient needed and that patients could have been exposed to a care injury during their work shift. It did not bring satisfaction when they left work with the feeling of not having been able to provide the patient with good and safe nursing care. They also pointed out that they had not been given time to “be new” but had to take on a responsibility that they had not expected right from the start. Sometimes they were also responsible for supervising nursing students and new staff when they did not feel secure in their profession. A lack of staff also meant that during certain work shifts only NGNs served, and then there was a feeling of being left alone to make decisions about patients' care.

…has been quite difficult, because many are bad. It's enough when you know that a patient is unwell and you're busy anyway. Then you have eleven more that need to be looked after. …feeling inadequate. So, you kind of think about what patient safety is…. Yes, you don't go home with a good feeling (5).

… hardly feel that all patients and do not know if all received exactly what they needed? No, a lot of people were undernourished and suffered care injuries during my shift.… pretty quickly I would also be a supervisor for new ones as well… is my introduction even finished… so reality shock… yes, there is a constant lack of time to get the work done. It's constant… a substandard work environment. It doesn't feel good (2).

#### 4.1.3 Lack of Support

Sometimes knowledge was acquired via a hospital admission interview with the patient, but the participants also needed to discuss the assessment with an experienced colleague who was sometimes not available. Attempts were made to seek out an experienced colleague or, alternatively, on one's own initiative, to read national guidelines. The NGNs agreed that they needed more introduction and support during the first 6 months. They also requested follow‐up, feedback, and feed forward from managers. A sense of security meant being able to consult someone about various considerations. NGNs felt that an RN assigned as support would be an advantage and follow‐up discussions with colleagues were better than with the head of department. The other NGNs who were in the same situation were a good source of support, and they offered the opportunity to seek security in each other. The NGNs, however, had to take responsibility from the beginning because there was a lack of staff.

… lack of staff, from the beginning … lack of support from the management… we were supposed to have more support as new… … we became a little bit, not alone, but we kind of had to go on our own two feet more than, you would have liked… But usually there has been a more experienced colleague who you could go and ask. Then you have been able to take help from the care handbook… I think, it would have been nice to have someone for the first six months to turn to, who might not be the manager but a nurse, someone with a little more experience. I know we have something called mentors, but I don't know if we have a specific person. It's like no one we've had contact with (5).

### 4.2 Challenges Lead to Professional Development

The NGNs described their first year in the profession as interesting and challenging. There was a demand for readiness for action, and this became even more evident because there were often staffing problems. This meant that they had to take their own initiative regarding which guidelines and routines applied in each care department. The NGNs described that each patient was a new challenge that also led to new knowledge and experiences. This helped to increase their confidence, but they expressed concern about not always being able to guarantee patient safety. Another challenge was to be flexible in the care of different patient groups and above all in connection with COVID‐19, where they were faced with dealing with different ethical situations. They felt that when they were faced with different challenges and stressful situations such as being the most experienced among colleagues, it led to positive stress and professional development. To meet these challenges, continued competence development with, for example, supervision of an experienced RN, opportunities to share experiences with peers in the same situation and support based introductory programmes are required.

#### 4.2.1 Take Your Own Initiative

After receiving their credentials, NGNs experienced pressure to live up to having knowledge related to patient care needs. There was a demand for readiness for action, and it became particularly noticeable when staffing was not complete during different work shifts. This meant that they had to take their own initiative early on because they were considered at certain times to be the most experienced. They expressed that they had to challenge themselves, and this led to increased self‐confidence but sometimes doubts as to whether this always contributed to safe nursing care. The NGNs considered that they had insufficient knowledge to be able to lead the nursing work and communicate with other health professionals who were involved in the patient's care. Some of the NGNs highlighted that there should be more focus on the nurse's future leadership in nursing education.

… even if you are under supervision for the first time, something happens when you have a degree… Now you must know this… a little pressure, that you have to deliver in some way. Because now all of a sudden, I have a sign that says I'm a nurse, and my patient doesn't know I got it yesterday (1).

… that you've had to step up because there's no staff, and we've also had a lot of people quit… you're standing here now and soon after a year, you're starting to be the one with the most experience on certain shifts and it doesn't feel safe for the patient. And it is also an adjustment. But then you may have challenged yourself a little, and that is probably also new, you may have gained a completely different self‐confidence during this time (5).

… quite young people who may not have the experience that you have when you're a little older… just to come out as a 25 year old and lead a team in a department. Too many people find it a difficult part of the profession… think there is far too little training in communication and leadership… (3).

During COVID‐19, NGNs were reassigned from their regular care wards to established care wards for the care of COVID‐19 patients only, to solve staffing problems. It meant new colleagues to deal with, and confusing information was given about hygiene routines. This led to the additional stress of having to stay up to date and be at the centre of events. However, it did mean a positive experience for their professional development as nurses.

… especially now with corona, and there are a lot of guidelines here and there. And now we do… like this week… now you don't have to wear a surgical mask, and now it was just a visor. A lot of information to take in simply when you are busy with yourself and everything else too… It was messy … been in three different departments and a lot of new colleagues, a lot of different personalities to work with and then being new…. and a new virus… (1).

…this thing about being thrown into a pandemic, that… I feel like I'm stepping up a bit, … some call it stressful, but an eventful job… I'd rather say that I'd rather call it (3).

#### 4.2.2 Complexity in Nursing

The NGNs described experiences of meeting seriously ill patients and their relatives and not having the knowledge and experience required to be able to answer their questions, especially during an ongoing pandemic. There were new challenges when it came to patients, drugs, and medical equipment. The NGNs described how they had to be flexible from day to day because they did not know which patients they had nursing responsibility for.

…. report that there is a new patient, may sound like it is very fragile. Then you can get a little nervous before you get to meet the patient… or perhaps have to give a new medicine that has never been given before, can also get nervous… or Modified Early Warning Scores (NEWS) check which doesn't look so good (1).…

…. then we are into medical technology, we are into medicines in general, what is included in the nursing profession, in that part. I still feel like a novice there, so it's more difficult the more you realize that this patient needs advanced care, and then it's tougher (3).

The complexity of care increased during the COVID‐19 pandemic, and NGNs also faced various ethical situations connected to patient care, where they were responsible for seriously ill and multi‐morbid patients as well as elderly patients and the contact with their relatives. Planning the patient care was different because of the barriers imposed by COVID‐19. NGNs felt that they could not meet the patient needs based on an ethical approach. This mainly concerned the care of elderly patients who were denied visits by their relatives during the pandemic. However, NGNs still felt that they had the opportunity to be responsible for patients who were not usually cared for in a ward, and this strengthened their professional development.

… the insecurity that you have felt, because no one knows anything, a new illness… But suddenly someone is sick, and someone is doing great… a lot of relatives who call … can't answer the questions, because I don't know… as well as in term of COVID‐19 care, we've had a lot of couples that have come. That is, older couples who have had home are… very difficult situations where people have had to lie down and be cared for together, have had to hold each other's hands, things that you will probably never forget… this ethical thing too, that they haven't been allowed to receive visitors (1).

… Many elderly people affected. Because, for one thing, they must be locked in the room, where you can keep an eye on them. Now we must go in and try to do everything we can at this moment and the brain has had to think one of which… you have to think like one step ahead (5).

#### 4.2.3 Share Experiences and Knowledge

The NGNs considered that they had chosen the right profession, they had passed their education and would be able to handle various challenges but expressed a need for introduction and competence development. Their participation in introductory programmes was perceived as being positive and developing, and being able to share knowledge and experiences with colleagues in the same situation made it therefore important to have physical meetings as opposed to digital ones. The NGNs highlighted that there are different requirements to meet digitally than in physical meetings, such as creating commitment, a sense of community, and getting in closer contact with each other. This meant daring to share and discuss both good and bad experiences during their first year. There were also suggestions from the NGNs about developing the introductory programme based on current conditions that arose in connection with COVID‐19.

I was one of five NGNs… my main support has been the colleagues and those who came together and know each other since the training, that we have been able to talk and ventilate with each other (5).

… incredibly valuable to get… meet people in the same situation and just get to share experience and talk to each other… these small moments between the lectures what have I succeeded or failed… The last one was in digital form… it is a more difficult forum to get the same commitment, and you don't have the same community, there should be physical meetings (3).

## 5 Discussion

This study describes NGNs' experiences in their first year. COVID‐19 reached pandemic levels 1 month after their graduation, significantly affecting their development in the profession. The participants emphasized that they felt inadequate and that they could not always guarantee safe nursing care. RN has an independent nursing responsibility for good and safe care, which means being able to work preventively and to be risk‐aware (Socialstyrelsen [The National Board of Health and Welfare] 2017). A problem that Gellerstedt et al. (2019) highlights is that experienced RNs leave their employment because they feel unable to provide safe nursing care that they consider patients need. This contributes to the fact that there is a shortage of experienced RNs, and the consequence is that there is a lack of role models for NGNs (Bahlman‐van Ooijen et al. 2023; Kox et al. 2020). Ball and Griffiths (2022) state that healthcare providers must have sufficient nursing staff to provide care safely and state that patients have the right to be treated with a professional standard of care by appropriately qualified staff. Sometimes there were staffing problems and then they had to be responsible for seriously ill patients, administer new drugs and manage advanced medical technology equipment. Ho et al. (2021) show that NGNs felt insecure whether they could ensure safe nursing care. Allvin et al. (2020) show that NGNs requested more knowledge related to managing health care and giving health promotion advice and recommendations when using e‐mail, telephone and digital technology solutions, and about interactions and side effects of various medicines. The same problem also appeared in specialist nursing and master's programmes (Taylor et al. 2020; Wangensteen et al. 2018). In both studies of Wangensteen et al. (2018) and Allvin et al. (2020), it was shown that NGNs rated their competence as low, when it came to interactions of various types of medications and Taylor et al. (2020) highlighted the need for further training in the use of electronic devices. The development of technology, places additional demands on NGNs and many countries are seeking to introduce advanced roles of nurses (Buchan et al. 2019). The NGNs in this study described experiences of inadequacy when they were responsible for multi‐morbid patients and there was a risk that the patient was exposed to risks in connection with their duties and sometimes, they sought knowledge on their own in manuals and the Handbook of Healthcare. Development of competence is a prerequisite for being able to make important decisions in the care of the patient (Allvin et al. 2020).

The NGNs expressed that early on they had to be responsible for prioritizing and assessing multi‐disease patients for whom they did not feel ready and had no experienced colleagues to consult. They experienced a lack of support during their first year in the profession. They had wanted an available experienced colleague to consult (Detlín et al. 2022; Ho et al. 2021) show that when NGNs were left alone, partly with increased responsibility and lack of support in decision‐making, this was experienced as a shock and several considered leaving the profession. They needed, according to Detlín et al. (2022) and Sterner et al. (2023) just a confirmation from an experienced colleague that they had understood the instruction correctly before performing a task and wanted a clinical mentor.

NGNs also experienced huge stress during their work and felt great responsibility leading to fear of failure, making it more likely they would leave the profession. Alrashedi and Boshra (2023) believe that more attention should be paid to stress in the workplace, as it can affect the NGN's ability to meet patients' needs and lead to a desire to quit. There is a lack of skills which means that NGNs do not receive sufficient supervision and support from experienced RNs during their first year. It is important to be aware of NGNs showing signs of stress and respond through an adapted workload, support from leadership and satisfactory collaboration with colleagues (Rudman et al. 2014). On the other hand, the possibility of venting with former fellow students in the same situation provided security during their first year. The job embeddedness seems, according to Ho et al. (2021), to be a useful lens that acted positively for the NGNs during their first year. This study showed that the NGNs experienced negative stress for not being able to guarantee safe nursing care, but positive stress meant that they did not consider leaving the profession without gaining more experience and then further training.

NGNs graduated a month before the outbreak of COVID‐19 and were not only challenged as being new to the profession but also being new in a pandemic. This brought additional challenges, such as more complex care and being able to handle different ethical situations in the care of seriously ill patients. Patients with COVID‐19 were isolated from their families' comforting presence to hold their hand or possibility to say goodbye. Also, the personal protective equipment and facial masks also constituted a barrier for the NGNs to show empathy and be able to communicate and interpret different signals from the patient, which was also confirmed by (Carnesten et al. 2025; Hofmeyer and Taylor 2021). There was an absence of clear guidelines regarding hygiene that could vary over time; no one knew anything and there was a risk to one's own health and that of the family. It emerged in this study that NGNs experienced frustration when hygiene guidelines were continuously changed, and according to Carnesten et al. (2022) they expressed feelings of confusion due to conflicting directives. Pellikka et al. (2024) highlight that it is important to provide a proper flow of information and ensure that the instructions are followed. They state that early information is a good way of reducing gossip. NGNs pointed to the additional requirements involved in handling more advanced equipment without direct supervision, which led, in the view of the NGNs, to patient safety being compromised. The NGNs in this study were reassigned to wards set up solely for the care of patients with COVID‐19. They felt insecure about the constant flow of new employees and sometimes they were the ones with the most experience. According to Pellikka et al. (2024) redeployment of personnel may mean an increased risk for patient safety, and it is important to ensure that competence levels are adequate. Nymark et al. (2022) also show that patient safety and quality of care were significantly worse, and missing care in wound care and basic nursing were reported. Although NGNs described chaotic situations and feeling stressed and worn out, they experienced professional development. This was also shown in a study by Danielis et al. (2021) that NGNs, who were recruited and reallocated to a COVID‐19 unit, experienced a mix of negative feelings in the early stages and at the end felt growth as a person, as a team, and as a professional.

There are staffing problems in healthcare and during certain duty shifts NGNs could be the most experienced. They were both responsible for supervising students and introducing new employees as well as being responsible for the patient's care. According to Eriksson et al. (2020) NGNs are not mature enough for the responsibility required of partly caring for the patient and partly supervising new colleagues. It can lead to feelings of uncertainty when they have not achieved security in their profession. Bahlman‐van Ooijen et al. (2023) show that heavy workload, staff shortages and no ideal ratio of patients to RNs contribute to a poor working environment. The NGNs in this study described that they lacked knowledge in leading the nursing work and pointed out that there should be more focus on it in the basic training for nurses. The education in leadership is lacking in basic nursing education and Francis‐Shama (2016) pointed out that it only comes at the end and should be a continuous theme throughout the programme. This study also proved that they needed competence development, but Lindfors et al. (2022) highlight that increased knowledge can not only be transferred in daily work but, according to Detlín et al. (2022), guidance is also needed on how the knowledge should be used in clinical practice. NGNs also felt less insecure about emergency situations. The NGNs in this study requested support during their first year in the profession in the form of clinical development programmes. Coffey et al. (2019) showed that education leads to improved job satisfaction, a sense of well‐being and increased pride in the nursing profession.

## 6 Limitations

The NGNs included in the study are from the same regional organization and attend the same Clinical Development Program (KUP I). This can be a limitation because other ways of organizing and structuring healthcare and programs can have other consequences for nursing responsibility and professional development, which might impact transferability. The plan was to conduct focus group interviews, but it had to be changed to group interviews as the number in the group changed due to illness or extra work shifts. This partly affected the size of the groups, and in one group there was an individual interview, and partly the length of the interview time (37 min). In the individual interview, there was no opportunity to share experiences with others, which contributed to the length of the interview being shorter. Despite this, the interview gave rich information. During the current pandemic, interviews were conducted digitally via Zoom. The digital interviews may have contributed to limiting the opportunity to share experiences, and nuances might have been lost.

The semantic level and critical reflective approach have been significant in facilitating a reflexive analysis in consideration of the interaction between the data, researchers and the social context in which the research was conducted (Braun and Clarke 2006, 2021). However, the data provides an understanding of NGNs' taking on responsibility for safe nursing and how COVID‐19 affected professional development during the first year of employment. We are aware that more research needs to be done on NGNs' responsibilities for safe nursing care as the understanding developed during this analysis can only be partial.

## 7 Conclusions

NGNs felt that challenges led to their development in the profession. The challenge for NGNs was that early in their entry into the profession, they had to take responsibility for their assessments of patients' care needs and safe nursing care. It was important to receive confirmation from an experienced RN about their planning of patients' care and highlighted that patient safety was sometimes at risk. In connection to COVID‐19, they were faced with new challenges, where decisions were to be made, which they did not consider having sufficient knowledge and experience to guarantee safe nursing care. The result of this study showed the importance of participation in Clinical Development Programs and meeting physically to share knowledge and experiences with each other, but that support of experienced RNs needs to be increased. Despite all NGNs emphasizing a continued future as RNs and planning a specialist education.

## 8 Relevance to Clinical Practice

It is important that NGNs are given the opportunity to be new and are offered continuous support and competence development, to strengthen their self‐confidence and security in the profession. The importance of being part of a team creates a sense of belonging in a context where knowledge and experience can be exchanged, which leads to professional development.

Healthcare policymakers, hospitals, and ward managers need to develop a long‐term strategy to support NGNs over a longer period of time through continuous development and not just during a 12‐month program. The program also needs to be clearly integrated into the regular ward work and involve experienced RNs in the competence development of NGNs.

## Funding

The authors have nothing to report.

## Disclosure

The study is qualitative, and this means that both results and discussion will be more comprehensive and contain more than 25 references.

## Conflicts of Interest

The authors declare no conflicts of interest.

## Data Availability

The data that supports the findings of this study are available from the corresponding author upon reasonable request.
